# Supplementary material for: Exploring the Black Box of an mHealth Intervention (LIFE4YOUth): A Qualitative Process and Outcome Evaluation of End-User Engagement
Source: Int J Environ Res Public Health. 2022 Oct 28;19(21):14022. doi: 10.3390/ijerph192114022 (PMC9653685; doi:10.3390/ijerph192114022)

Figure S1: Screenshots of the LIFE4YOUth intervention

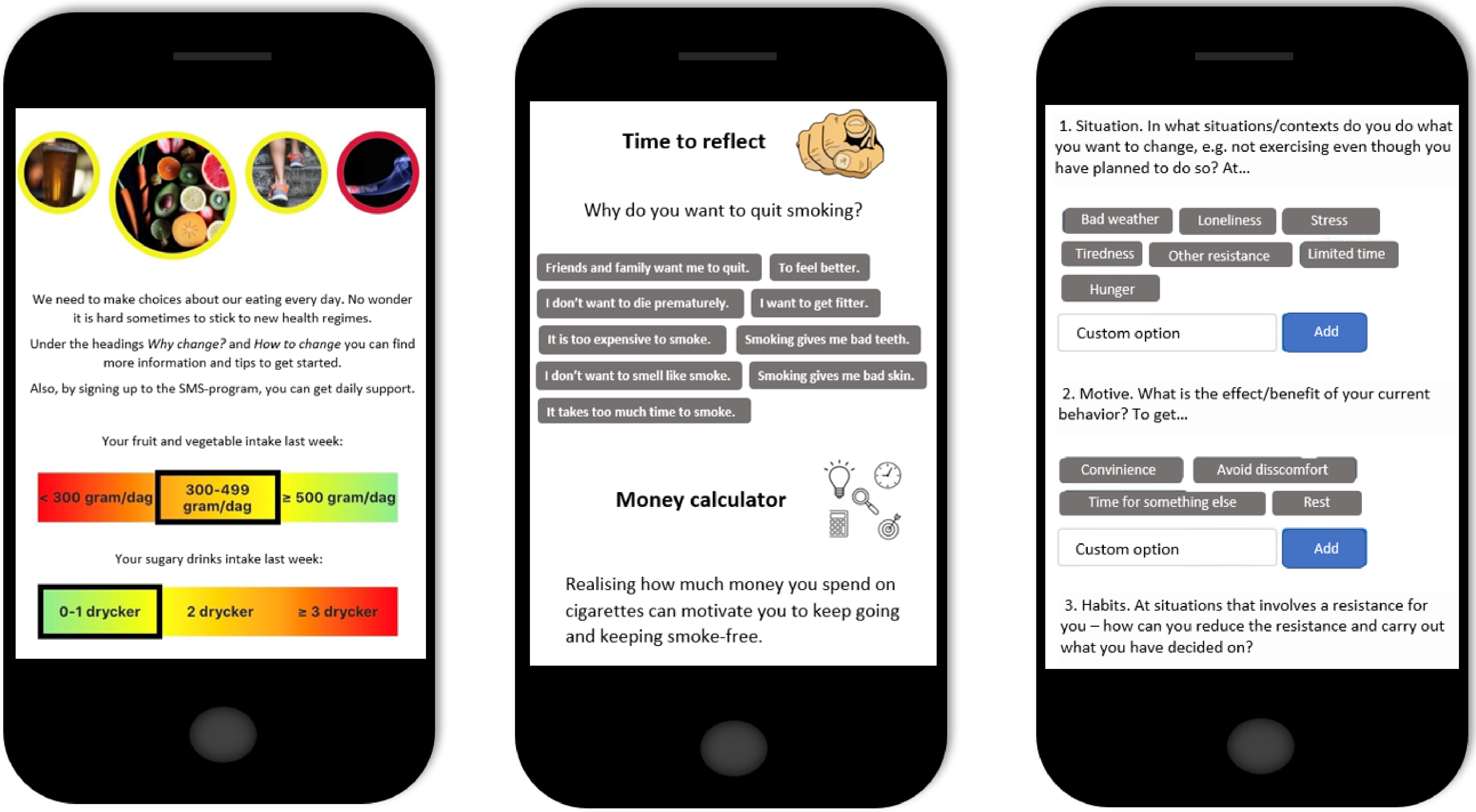

Supplement: Supplementary file 1 [file ijerph-19-14022-s001.zip › ijerph-1973814-supplementary.pdf]
